# Discovering Genome-Wide Tag SNPs Based on the Mutual Information of the Variants

**DOI:** 10.1371/journal.pone.0167994

**Published:** 2016-12-16

**Authors:** Abdulkadir Elmas, Tai-Hsien Ou Yang, Xiaodong Wang, Dimitris Anastassiou

**Affiliations:** 1 Department of Electrical Engineering, Columbia University, New York, New York, United States of America; 2 Department of Systems Biology, Columbia University, New York, New York, United States of America; University of Texas Rio Grande Valley, UNITED STATES

## Abstract

Exploring linkage disequilibrium (LD) patterns among the single nucleotide polymorphism (SNP) sites can improve the accuracy and cost-effectiveness of genomic association studies, whereby representative (tag) SNPs are identified to sufficiently represent the genomic diversity in populations. There has been considerable amount of effort in developing efficient algorithms to select tag SNPs from the growing large-scale data sets. Methods using the classical pairwise-LD and multi-locus LD measures have been proposed that aim to reduce the computational complexity and to increase the accuracy, respectively. The present work solves the tag SNP selection problem by efficiently balancing the computational complexity and accuracy, and improves the coverage in genomic diversity in a cost-effective manner. The employed algorithm makes use of mutual information to explore the multi-locus association between SNPs and can handle different data types and conditions. Experiments with benchmark HapMap data sets show comparable or better performance against the state-of-the-art algorithms. In particular, as a novel application, the genome-wide SNP tagging is performed in the 1000 Genomes Project data sets, and produced a well-annotated database of tagging variants that capture the common genotype diversity in 2,504 samples from 26 human populations. Compared to conventional methods, the algorithm requires as input only the genotype (or haplotype) sequences, can scale up to genome-wide analyses, and produces accurate solutions with more information-rich output, providing an improved platform for researchers towards the subsequent association studies.

## Introduction

**Fig 1:**
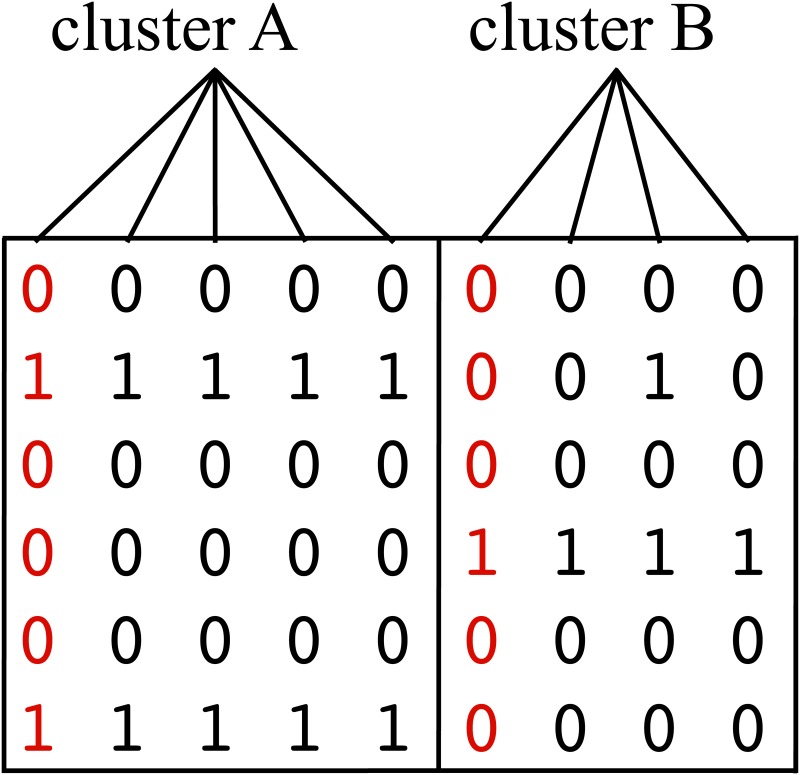
Example for clustered SNPs. 2 tag SNPs are selected by clustering the 9 SNPs according to the genotypes from 6 individuals. As presented in Fig 1, the SNPs can be grouped into two groups by their patterns of the genotypes. The SNP that are marked in the red color are selected as a representative SNP, or tag SNP of each of the two clusters.

The basic unit of genetic variation is the *single nucleotide polymorphism* (SNP) which refers to single base-pair changes in the DNA sequence of an individual’s chromosome [1, 2]. SNPs are located in various sites of a pair of near-identical chromosomes. Most experimental techniques can determine an unordered pair of allele readings for each SNP site to build an individual’s genotype sequence. Given a population of individuals, a high degree of correlation is observed among the nearby allelic variations (linkage disequilibrium, LD), whereby most of the SNP sites convey redundant information and may be omitted for cost-effectiveness during genotyping [3]. For this, many studies have aimed to find such “*representative*” SNPs (*tag SNPs*) that can provide sufficient information about their nearby variants that are not genotyped. More formally, given the genotype sequences consisting of *N* SNPs obtained from a population of *P* individuals, the number of SNPs that capture the genomic diversity (haplotype diversity) in that population may be greatly reduced to a subset of representative SNPs where each such SNP will represent a cluster of redundant variants. This may be described by a clustering problem where *N* SNPs are divided into a number of distinct clusters according to some measure of similarity between the observations ***s***_*i*_, *i* = 1, …, *N* taken over *P* samples, i.e., ***s***_*i*_ = [*s*_*i*_(1), …, *s*_*i*_(*P*)]. Each cluster is characterized by the similarity (redundancy) between its observation vectors ***s***_*i*_ and a centroid vector (tag SNP) will be the “representative” of that cluster (Fig 1).

The SNP tagging approaches can be categorized into block-based and block-free methods. The block-based methods exploit prior information about haplotype block structures [4], and identify the optimal subset of SNPs (i.e., tag SNPs—also commonly referred as *haplotype tagging SNPs (htSNPs)*) in order to capture most of the haplotype diversity in a given block [5, 6]. However, block-based methods may suffer from inaccuracies caused by the block partitioning results [7]. A potentially better alternative may be (block-free) genome-wide methods. In genome-wide approaches two strategies are generally used for selecting representative SNPs, i.e., haplotype reconstruction-based methods and LD-based methods. The former involves a series of post-analyses (wrapper methods) for refining an initial inference through improving the haplotype reconstruction accuracy. Given a particular solution the representative (tagged) SNPs are considered as informative sites, and the allelic information on non-tagged sites (i.e., haplotypes) are predicted by a machine learning algorithm. In this methodology, the accurate prediction of haplotypes indicates that the given tag SNPs contain enough information about other SNPs and are sufficient for genotyping [8]. An optimization procedure (wrapper method) is run by employing the given informative SNPs until a desired accuracy level is obtained. Various strategies can be used for the initial selection of informative SNPs, e.g., regression-based [8], correlation-based [9, 10] etc. One limitation of wrapper-based methods is though the reconstruction scheme, which entails considerable computational complexity and can be impractical for high-throughput data. On the other hand, LD-based methods aim to identify regions of SNPs with high linkage disequilibrium through the discovery of recombination hotspots [4, 11]. In genetic studies it has been observed that there is a block-like structure between two adjacent hotspots where limited (or no) recombination events occur, and the SNPs within the block are often inherited together (i.e., linked) carrying redundant information [12, 13]. In other words, such a block possesses very low haplotype diversity across the population and the information carried by respective SNPs becomes highly redundant [5], suggesting that some subset of the SNPs can be sufficient to represent the diversity of the haplotype patterns observed in this block [6]. In a large genomic region the set of linked SNPs (blocks) can be identified through estimating the structure of haplotype blocks, e.g., by maximizing some measure of correlation (LD) among the variants [14, 15]. Then the SNPs representing each block can be picked in numerous ways (e.g., greedy forward-selection algorithm [16], LD-based or wrapper optimization based selection algorithms [17] etc.).

SNP tagging approaches are conventionally cast as a two-phased optimization problem, i.e., inferring highly-linked SNP sets (or haplotpye blocks), and selecting representative SNPs based on the inference result. Under such framework various algorithms have been proposed, including forward/backward selection based greedy algorithm [16], greedy pair-wise selection/prioritization algorithm [18], mutation/survival based genetic algorithm [19], clustering/elimination based greedy algorithm [17]. Another method is to use PCA to identify the principal components in a SNP data sets, but PCA requires the principal components to be mutually orthogonal, also PCA is computationally infeasible for large data sets [20]. In this paper, we introduce a block-free solution that can jointly (and more accurately) estimate the putatively-linked SNP sets and their tag SNPs, through exploiting a measure of mutual information estimated among the SNPs and the sets of linked SNPs for mapping the multi-marker associations. Information theoretic approaches have been used in earlier studies as a base for quantifying haplotype diversity and SNP selection by maximally retained information content [21–23].

In this study, the mutual information is used to measure the degree of association between the individual variants and the sets of linked variants. For this, we employed the computational algorithm in [24] presented for the analysis of gene expression data sets, which is an unsupervised iterative approach that converges to the core (“heart”) of coexpression of a given arbitrary gene in the data. When applied to SNP tagging problem, the method discovers distinct patterns of mutually associated SNPs through iteratively updating each pattern, and converges in a reasonable time that is proportional to data size. The details are given in the next section.

## Materials and Methods

### The attractor metaSNP

A set of linked SNPs in a high-LD block can be thought of as a cluster of variants of high mutual association. Of practical interest in this cluster may be to find the most representative SNP (tag SNP). For this, pair-wise or multi-locus LD metrics are widely used for quantifying the association between SNPs [16, 25–27], and the representative SNPs are selected accordingly. However, such pair-wise (or multi-locus) analyses among the individual (or subsets of) SNPs may fail to incorporate the broad association of each SNP with the “*heart*” of the cluster/block due to the structural changes of the chromosome, such as chromosomal crossover [13]. In this sense, we can define a consensus “*metaSNP*” (defined below) to represent the broad variation in the cluster, e.g., some sort of average value of the known allelic information over the variants. Then we can simply rank all individual SNPs by their “pair-wise” association with that metaSNP where the few SNPs with the largest association measure may represent the core of the underlying linkage disequilibrium block.

When analyzing gene expression data, a metagene is a hypothetical gene whose expression level is a weighted average of the expression levels of the particular individual genes. In [24], the authors present an iterative (“attractor”) algorithm, in which each “seed” gene leads to a successive sequence of metagenes. If the seed gene is a member of a co-expression signature, then this process converges to an “attractor metagene” representing the heart of co-expression. Although the algorithm is unsupervised, attractor metagenes proved to represent important biomolecular events and were used successfully for prognostic models for breast cancer [28, 29]. The algorithm has also been used to define signatures of mutually associated features (“attractor metafeatures”) from data other than gene expression, such as methylation and protein activity levels [30]. The attractor program is available as an R package under the Synapse ID *syn1446295*, and has also been adopted as a function (*metafeatures*) in MATLAB’s bioinformatics toolbox [31].

In the case of SNP data, a “metaSNP”, whose value is defined as a weighted average of the tri-level values of particular individual SNPs, cannot be thought of as hypothetical SNP because it is continuous-valued. Nevertheless, the above methodology is still directly applicable, and the resulting “attractor metaSNP” can represent a particular haplotype block (corresponding to a cluster of SNPs) due to high-LD, indicating the presence of a joint (rather than pair-wise) linkage. This is biologically relevant since the co-inheritance of SNPs naturally occurs in contiguous stretches, and this “joint” association is gradually degraded with the generational age due to being broken apart by recombination events [32]. From this point of view, a given cluster’s attractor metaSNP could be a suitable proxy encoding the broad allelic variation in the corresponding high-LD SNP region, whereby a tag SNP can be selected among the essential contributors of that metaSNP (i.e., the few SNPs with the largest association value). Therefore, we employed the aforementioned algorithm (Synapse ID *syn1446295*), and modified it for finding the attractor metaSNPs where the descriptions are given as follows.

### Attractor metaSNP estimation for a particular seed

Without any prior information, the problem of finding tag SNPs in a given genomic region can be cast as finding a number of distinct metaSNPs which can represent all common variations in layers (blocks) in the respective region. Given *N* SNPs and *P* samples, the vector with the values of a metaSNP M is the weighted average of all SNP vectors, ***s***_*i*_ ∈ {0, 1, 2}^*P*^, *i* = 1, …, *N*, characterized by the set of weights ***w*** = [*w*(1), *w*(2), …, *w*(*N*)], i.e., $M=\sum_{i=1}^{N}w\left(i\right)s_{i}$. Each individual SNP in the data can play the role of a “seed”, and can be processed using the above algorithm to estimate its attractor metaSNP and the associated weight vector, as follows.

When the *k*-th SNP is used as seed, the corresponding metaSNP is initialized by using a set of (trivial) weights specific to that seed, i.e., a length-*N* vector of zeros except for the *k*-th element which is 1. This choice initializes the metaSNP $M_{k}$ as being equal to ***s***_*k*_. In the next step, the pair-wise associations between each SNP vector ***s***_*i*_ and the metaSNP is calculated by the similarity metric

$$J\left(s_{i},M_{k}\right)=I^{\alpha }\left(s_{i},M_{k}\right),$$ (1)

where *I*(*x*, *y*) ∈ [0, 1] is a normalized estimation of the mutual information [33] between the two random variables *x* and *y*, and *α* is a nonnegative power exponent that shapes the similarity metric in a nonlinear manner pushing smaller values of the normalized mutual information closer to zero. We use *α* = 5, as in [24]. This measure is used for the updated set of weights in the next iteration, i.e., $w\left(i\right)=J(s_{i},M_{k}$), and the new estimation of the metaSNP is obtained by the updated weights. After iterating several times, the weights tend to stabilize whereby the convergence is determined, i.e., the algorithm stops when the norm of difference between the two consecutive weight vectors drops below a certain threshold, at which point the iterative process is assumed to have converged to the attractor metaSNP (Algorithm 1).

**Algorithm 1** Attractor metaSNP

1. Start with the *k*-th SNP as seed.

2. Calculate the pairwise associations (weights) between the *k*-th SNP and all other SNPs, i.e., *w*(*i*) = *J*(***s***_*i*_, ***s***_*k*_), *i* = 1, …, *N*.

3. Estimate the metaSNP by taking the weighted average of all SNP vectors, i.e., $M_{k}=\sum_{i=1}^{N}w\left(i\right)s_{i}$.

4. Calculate the (multi-locus) associations between the metaSNP $M_{k}$ and all other SNPs, i.e., $w\left(i\right)=J\left(s_{i},M_{k}\right)$, *i* = 1, …, *N*.

5. Repeat the steps 3-4 until the two consecutive weight vectors obtained at the 4th step are very similar, i.e., $\sqrt{\sum_{i=1}^{N}{\left(w^{new}\left(i\right)-w^{old}\left(i\right)\right)}^{2})}<\epsilon$, or a predefined maximum number of iterations is reached.

6. Return the attractor ***w***, and the metaSNP $M_{k}$.

### Finding all attractor metaSNPs

We can do an exhaustive search by applying the attractor algorithm for all *N* seeds. In that case, we will find a limited number of attractor metaSNPs, each of which has multiple “attractee” seeds (i.e., a seed that converges to a “particular” attractor) [24]. It would suffice to constrain the “set of seeds to be processed” to a subset of {1, …, *N*} such that it will consist of only one attractee seed (from the equvalent attractees) to efficiently result in the same set of attractor metaSNPs. To overcome such complexities, we developed a sliding-window based heuristic using the two objectives described below, which we observed that do not compromise performance.

- First, for a given seed SNP, we will constrain the weighted average (metaSNP) and the association calculations to the seed’s “genomic neighborhood” with X local SNPs (we used X = 10,001), i.e., use $i=k-\frac{X}{2},\;\ldots ,\;k+\frac{X}{2}$ in steps 2-4; otherwise, using all N SNPs at the repeated steps 3-4 in Algorithm 1 can be computationally prohibitive for large N, making the algorithm intractable (e.g., the 1000 Genomes Project [34] provides the genotype data with N = ∼84 million variants).
- Second, to estimate all attractor metaSNPs in the data efficiently, we will reduce the number of possible seeds by evaluating their potential to be an attractee seed. For every SNP (*k* ∈ 1, …, *N*) in the data, we quickly estimate a “short-attractor” (consisting of 101-SNPs) by using the Algorithm 1 with $i=k-\frac{101}{2},\;\ldots ,\;k+\frac{101}{2}$ in the steps 2-4, and call it an attractee seed if the 5th largest weight in the converged ***w*** is larger than 0.5; otherwise, discard the seed.

Given the above definitions, the sliding-window heuristic is summarized in Algorithm 2.

**Algorithm 2** Finding all attractors

1. Estimate all attractee seeds having 5th largest weight in its short-attractor ≥ 0.5.

2. Run the genomically-localized program for every attractee seed reported from the short-attractors.

3. Return all attractors estimated in step 2.

### Selecting and ranking tag SNPs

Since the weights used for the attractor metaSNPs are equal to their associations with the individual SNPs, it is straightforward to select the tagging SNP as the one with the largest weight. In case of multiple (co-)top-ranked SNPs with identical (largest) weights, we choose the one that is located closest to the median of their genomic positions. After identifying all tag SNPs, one can order them to assist the selection of informative tag SNP subsets for various genotyping needs (see Results for the analyses of genotype coverage obtained by different choices of tag SNPs). The information value of a tag SNP may correlate with the “strength” of its attractor measuring the degree of association in the attractor’s top SNPs. To favor a tag SNP with strong mutual associations in its top-ranking variants, we define the strength *S* of an attractor as “the (unnormalized) mutual information between the *n*-th top SNP and the attractor metaSNP”. By default, we set *n* to 10 as it leads to good performance in most data sets.

## Results

In this work we conducted a series of experiments on widely-used SNP data sets to assess the proposed method’s performance in terms of efficiency in SNP tagging. We compared our results with the state-of-the-art algorithms designed for the same task on the relevant data sets.

### Data sets

#### HapMap

We used the trio genotype data sets from HapMap’s ENCODE project [35, 36] belonging to 30 trio families from the CEU population. We focused on four genomic regions ENm013, ENm014, ENr112 and ENr113, where the largest (ENr113) contain 2486 SNPs covering ∼500 kb region of the Chromosome 4 corresponding to an average marker density of 1 SNP per ∼0.2 kb. The details of the data sets are listed in Table 1. For a wider application in HapMap, we also used the genotypes corresponding to human Chromosome 22 [37] consisting of 60 trio samples from the CEU population. All genotype data sets come with missing values for certain SNPs and samples. We used IMPUTE2 algorithm [38] to impute missing values by employing as the reference panel the genotypes from 1000 Genomes Project [34, 39]. In addition to imputed data, we run the algorithms using the raw (unimputed) format of the data sets to see their performance under missing data condition. We also tested the algorithms’ performance for tagging SNPs in haplotype data sets. For this we used the same ENCODE genotypes and phased the corresponding haplotypes by using IMPUTE2 algorithm. Due to limitations in inference accuracy we only used the confidently-phased SNPs, which reduced the data sizes of ENm013, ENm014, ENr112 and ENr113 to 1626, 1712, 1366 and 1998 SNPs, respectively.

**Table 1:** Details of HapMap data sets used in this study.

Dataset	Chr. no.	No. of SNPs	Marker sparsity (bases)	No. of samples
ENm013	7	2069	241.5	90
ENm014	7	2232	222.7	90
ENr112	2	1505	332.1	90
ENr113	4	2486	200.9	90
Chr22	22	20108	1741.3	165

#### 1000 Genomes Project (1KGP)

In addition to HapMap database, we tested the algorithms on the genotype data from the recently catalogued 1000 Genomes Project [40]. These genotypes are constructed based on a large group of individuals from multiple populations, combined with genotype imputation on the variants not covered by sequencing reads, which obviated the imputation step in our analyses. In this work, we used the genotype data from the latest release consisting of 84.4 million variants built by the 2,504 samples from 26 populations [41, 42]. We tested our algorithm for the whole-genome data by processing one chromosome at a time to scrutinize the tagging results specifically for each chromosome. As a result, we built the 1000 Genomes TagSNP database [43] displaying the tag SNPs, and the SNPs they tag, provided with the respective joint association measures (i.e., the attractors depicting the multi-locus LD maps).

### Performance evaluation

As the performance metric we used “*coverage rate per tagged SNPs*” to represent the genomic diversity (or genotype diversity) captured by a given choice of tag SNPs. The *coverage rate* (*R*) can be defined as “the ratio between the maximum number of genomic sequences covered (*G*_*i*_) and the total number of samples (*P*)” for the given choice of tag SNPs [17], i.e.,

$$R=\frac{\sum_{i}^{t}G_{i}}{P}$$ (2)

where *t* is the number of genomic patterns observed on the given selection of tag SNPs. In genotype data, those patterns are the distinct sequences of genotypes, each consisting of the three-level genotype values of an individual in the selected tag SNPs, i.e., 0 encodes for the reference homozygous genotype, 1 encodes for the heterozygous genotype, and 2 encodes for the alternate homozygous genotype. For example, assume that 5 samples (I-V) are genotyped on four SNP loci and the corresponding sequences are “2111”, “2200”,“2201”,“2110”, and “2200”. If the first two SNPs are tagged, there will be only two genotype patterns “21” and “22” observed on the samples I,IV and II,III,V respectively. The pattern “21” can represent the genotype sequences “2111” (I) and “2110” (IV), each belonging to exactly 1 individual, so that the maximum coverage of this pattern is *G*_1_ = 1. The second pattern “22” represents the genotype sequences “2200” (II,V) and “2201” (III), where the sequence “2200” covers 2 individuals (samples II and V) and the maximum coverage is *G*_2_ = 2. Using Eq (2) the coverage rate *R* is calculated as (*G*_1_+*G*_2_)/*P* = (1+2)/5 = 0.6. Intuitively, an optimal tagging approach should find a subset of SNPs possessing larger number of genomic patterns with perhaps a broader coverage obtained by each pattern, e.g., the last two SNP loci in this example will result in 4 distinct patterns covering 100% of the individuals (*R* = 1). *R* is a versatile metric that can be readily used in the haplotype data to represent the “*haplotype diversity*” captured by a set of tagged SNPs [17].

In genotype data sets, we compared our results with the state-of-the-art algorithm Tagger [18] which is employed by HapMap database as a SNP tagging tool. Tagger is a block-free (LD-based) approach that offers multiple LD measures for optimal SNP selection, and is robust for tagging SNPs in samples from multiple populations [44]. The version we experimented is maintained by Haploview (4.2) software [45]. For the phased haplotype data, we used another LD-based approach, ER algorithm [16], which is the most relevant work to the theoretical part of our study. ER algorithm uses information theory to define a multi-locus LD measure which is estimated by a form relative entropy calculation among the variants, and can only work with haplotype data.

We run the metaSNP method versus Tagger and ER on four ENCODE regions and Chromosome 22 in HapMap, and obtained the ranked estimates of tag SNPs solved by each algorithm. For the 1KGP data, we compared our predictions with those of the Tagger obtained for several short (50,000 SNPs) segments of the Chromosome 22, due to limitations pertaining the Tagger algorithm. In all simulations we used the recommended default settings of algorithms unless otherwise noted. We demonstrated the coverage rate (*R*) obtained by the top-*c* tag SNPs of a solution, whereby different values of *c* are used to illustrate the tradeoff between the coverage rate (*R*) and the genotyping cost (*c*), which is the number of SNPs that are tagged (for maximal coverage) in the given genomic region.

We compared the coverage rates that were achieved by metaSNP and Tagger at a specific cost using the z-test as well as at different costs using the Kolmogorov-Smirnov test. To assess the effect of increasing the cost, we tested the results at different costs using McNemar’s test. To compare the minimum numbers of the tag SNPs that were identified by the two methods to reach >95% coverage, we tested the difference of the tag SNP numbers using the t-test (S1 Table).

### Genotype data sets from several ENCODE regions and the human chromosome 22

**Fig 2:**
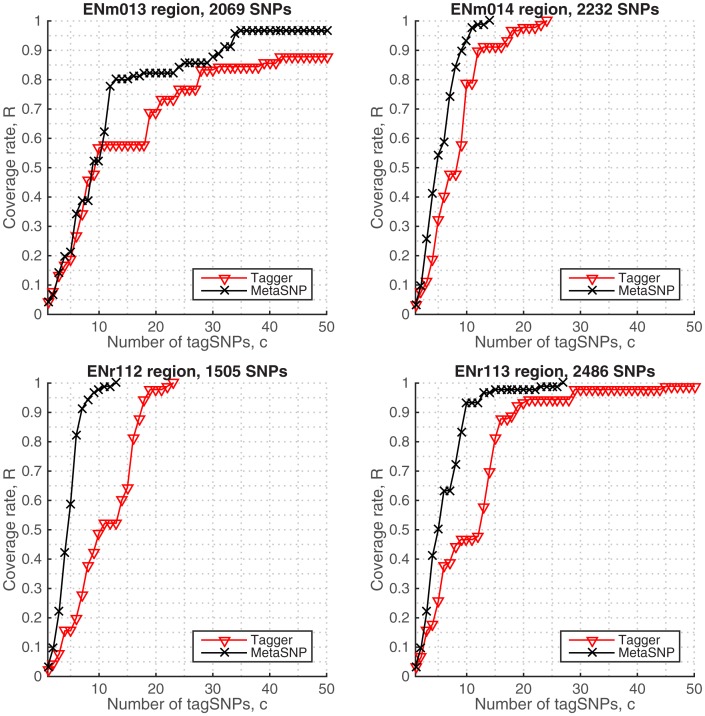
Coverage rates in imputed genotypes, HapMap.

It is seen from the Fig 2 that the algorithms perform favorably in ENCODE data by reaching a coverage rate of 90% within the ∼20 tagged SNPs. In all genomic regions the curves tend to saturate after ∼15 tag SNPs which corresponds to 90-100% coverage. MetaSNP significantly outperforms Tagger when the genotyping cost is low. The two algorithms perform equally well when the price range is high (Table 2). From the plots we can say that a coverage rate of 95% will be a good tradeoff since the genotyping costs exponentially increase after this rate. In this value, metaSNP algorithm is more cost-effective than Tagger (Table 3). The comparison of the discovered tag SNP sets are given in S2 Table.

**Table 2:** Coverage rates in imputed genotype data, HapMap.

	cost (c)	ENm013	ENm014	ENr112	ENr113
Tagger	15	0.58	0.91	0.64	0.81
metaSNP	15	0.80	1.00	1.00	0.98
Tagger	20	0.69	0.98	0.98	0.93
metaSNP	20	0.82	1.00	1.00	0.98

**Table 3:** Minimum number of tag SNPs that reach *>95%* coverage in imputed genotype data, HapMap.

	ENm013	ENm014	ENr112	ENr113
Tagger	>50	18	19	29
metaSNP	34	11	9	13

**Fig 3:**
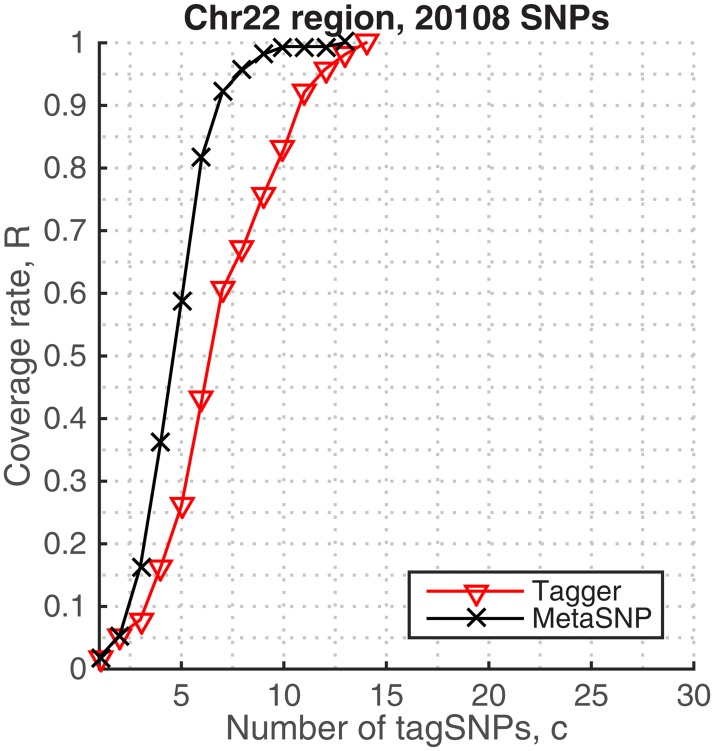
Coverage rates in imputed Chromosome 22 genotypes, HapMap.

The algorithms achieved similar behavior when tagging SNPs chromosome-wide. Fig 3 shows the coverage rates in Chromosome 22, for different choices of the tag SNPs. Notably, both metaSNP and Tagger algorithms can cover 90% of the genomic diversity by only 7 and 11 tag SNPs, respectively, and reach 99% in 10 and 14 tag SNPs, respectively. However, in the chromosome level, we can say that metaSNP algorithm outperforms at all genotyping costs in terms of coverage rates. The numbers of tag SNPs that are required to cover the genomic regions also summarize their haplotypic structure. The metaSNP method identified the same number of tag SNPs that describes the ENr113 region of chromosome 4 and chromosome 22, which reflects that chromosome 22 has a lower density of SNP than chromosome 4 [46].

### Haplotype data sets from ENCODE regions

**Fig 4:**
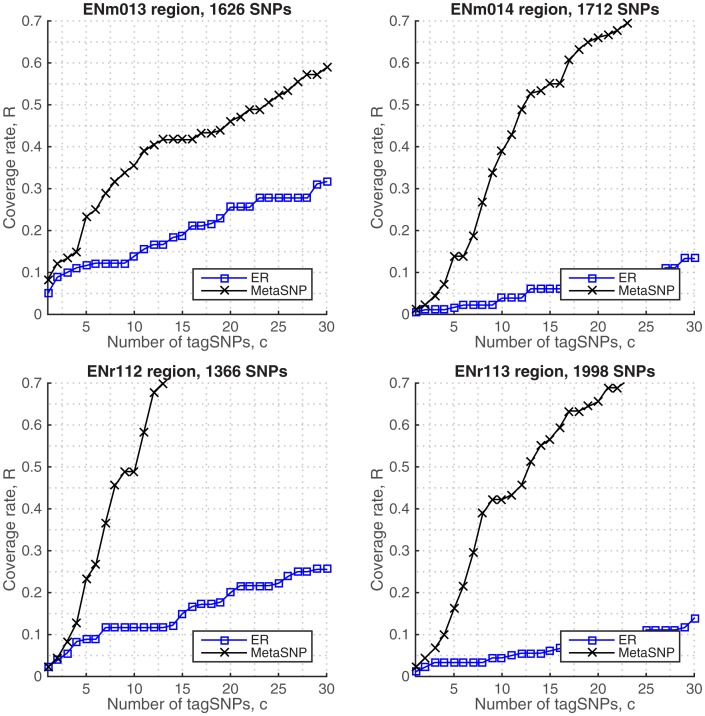
Coverage rates in phased haplotypes, HapMap.

The proposed approach can readily employ haplotype data and perform SNP tagging. We used the phased haplotypes obtained from the imputed ENCODE data and tested the performance of metaSNP against the ER algorithm. In these plots, the coverage rates are calculated from the haplotype sequences by using Eq (2) for the given selection of top-*c* tag SNPs. In Fig 4, it is seen that metaSNP algorithm significantly outperforms ER at all genotyping costs after 5 tag SNPs. This is due to multi-locus LD measure of ER which relies on a predetermined constant to weight the diversity and association of the SNPs, may accurately find low-LD tag SNPs and capture the haplotype diversity in sparse SNP regions, however it becomes ineffective when the data set deviates from the assumption. This can be observed in the denser (high-LD) SNP regions (i.e., ENr113 and ENm014 plots), where increasing the number of tag SNPs fails to incorporate a relative gain in coverage.

### Missing genotype datasets from ENCODE regions

**Fig 5:**
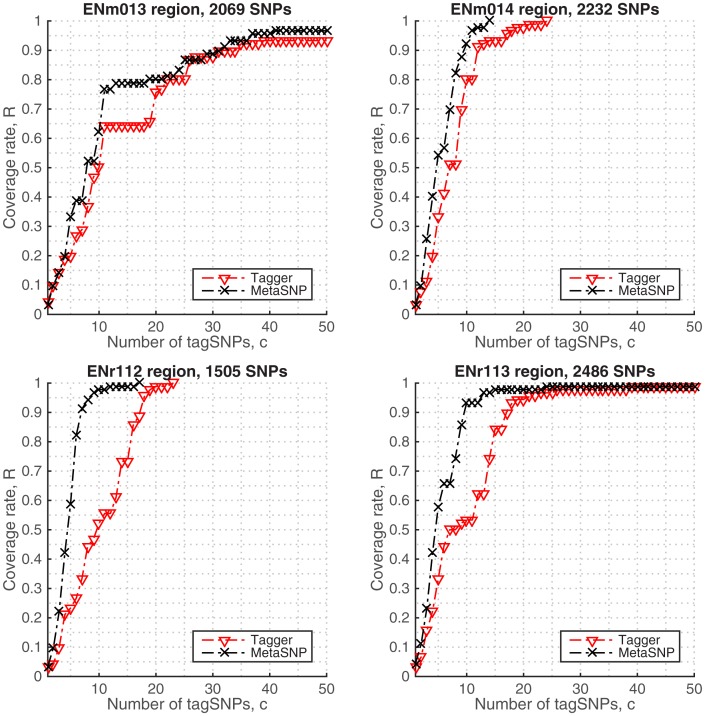
Coverage rates in missing genotypes, HapMap.

We tested algorithms under missing data conditions. In this experiment, the coverage rate *R* cannot incorporate missing alleles since those sites are typed with a nonspecific value. One can use the imputed genotype values corresponding to a given choice of tag SNPs. However, we simply ignored those missing loci in calculating *R* to avoid any imputation bias which may affect the algorithms, i.e., in Eq (2) we excluded missing sites from the analyses when determining the patterns and the number of samples they cover. Fig 5 displays the performance of metaSNP against Tagger in the raw ENCODE genotypes, where metaSNP have similar or better rates (Table 4). From the plots, we can say that the missing data have little or no impact on both algorithms where they can accurately perform SNP tagging on all sites and capture the majority of genotype diversity on “non-missing” loci.

**Table 4:** Coverage rates in missing genotype data, HapMap.

	cost (c)	ENm013	ENm014	ENr112	ENr113
Tagger	15	0.64	0.93	0.73	0.84
metaSNP	15	0.79	1.00	0.99	0.98
Tagger	20	0.76	0.98	0.99	0.94
metaSNP	20	0.80	1.00	1.00	0.98

### Genotype data sets from 1000 Genomes Project (1KGP)

Compared with HapMap, the main advance in 1KGP data is the greater SNP density (∼50 times denser) obtained from a rich multi-population sample set (2,504 individuals). Combined with genotype imputation this results in many variants with (ignorably) low frequencies in the overall population, e.g., the variants carrying only the reference homozygous genotype for all samples in the populations. In terms of tagging costs, as one can expect, compared to HapMap relatively more tag SNPs are required to reach the same coverage rate in the larger population (i.e., to cover more samples).

**Fig 6:**
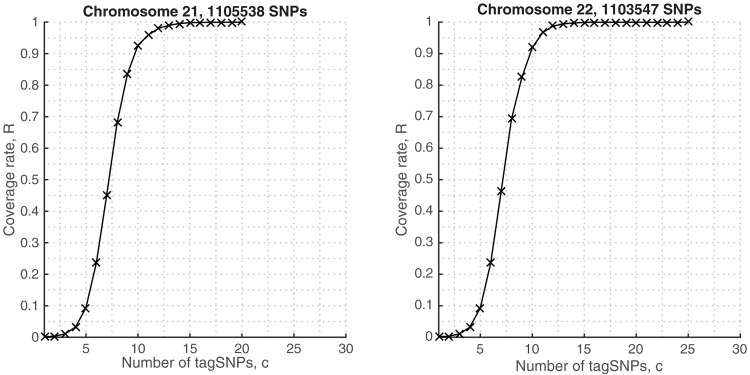
Coverage rates in 1KGP genotypes, Chromosomes 21 and 22.

First, we evaluated our method in the Chromosome 22 genotypes to provide a baseline tagging results against the metaSNP’s HapMap predictions. Then we used the Chromosome 21 genotypes, the shortest autosome, to further scrutinize our predictions. It is seen in Fig 6 that the algorithm finds 11 tag SNPs that sufficiently cover 95% of the samples and 25 tag SNPs for the full coverage in Chromosome 22. It performs better in Chromosome 21 genotypes, requiring less number of tags (20) for the full coverage of the samples. In both results the tagging SNPs are spread across the chromosome (S1 Fig).

**Fig 7:**
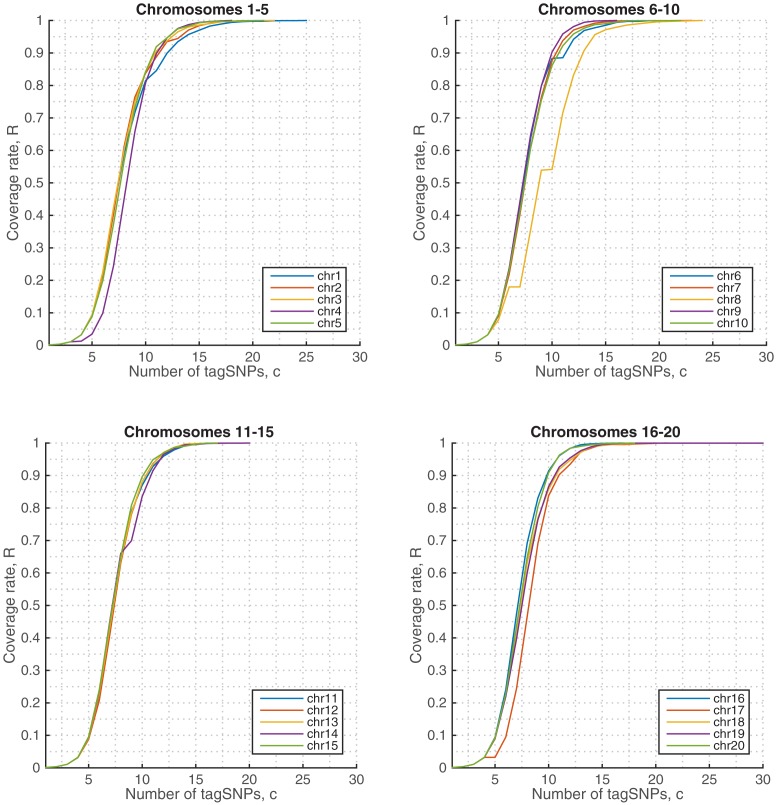
Coverage rates in 1KGP genotypes, Chromosomes 1-20.

We observed similar performance in other chromosomes as well. Fig 7 displays the coverage rates in the remaining 1KGP chromosomes, where we see that the algorithm is able to discover only ∼15-20 tag SNPs for the full coverage of the samples.

#### Comparison with Tagger

**Fig 8:**
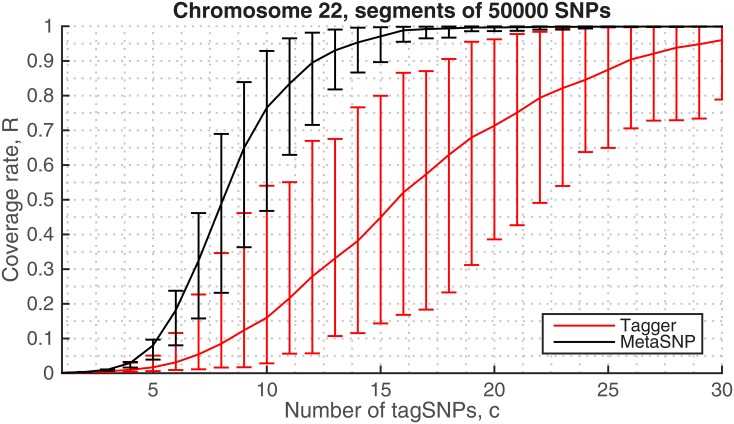
Comparison of the coverage rates in 1KGP chromosomal segments. The average performance curve is estimated from 20 consecutive blocks of 50,000 SNPs. For each given cost *c* the error bars represent the best and worst coverage rates, i.e., confidence intervals, obtained from the tagging results in different blocks.

As a performance comparison, we tested Tagger algorithm in the same 1KGP data sets. Since Tagger is based on the (offline) pair-wise analysis of all SNPs, the limitations occur due to memory use. To overcome this and to set out a fair comparison, we divided the data into several chromosomal segments (each containing 50,000 SNPs) then processed the individual segments. Fig 8 displays the performance curves of both algorithms averaged over these segmented data sets. We can say that, approximately 19 tag SNPs estimated by the metaSNP approach are sufficient to capture all genotype diversity in an arbitrary 50,000-SNPs genomic region. In contrast, the Tagger’s estimates can reach a similar performance at the cost of > 30 tag SNPs.

We note that the performance of our algorithm improves with the increased data size, i.e., given more variants residing in the neighboring genomic distances. This can be observed from Fig 8 as the confidence upper-bounds in the metaSNP’s coverage rates overlaps with the results in Fig 6 (Chromosome 22) which is based on the whole chromosome data.

### Phenotype association

In addition to the coverage rate, we assessed the tagging performance of the proposed method by comparing the genotype-phenotype association of the tag SNP and the SNPs that were correlated with the tag SNP.

Using the SCRIME package in R, we synthesized a 100-SNP array data set of 1,000 samples with a phenotype which is associated with the genotype of 3 designated SNPs with a three-way interaction as well as 1,000 samples without this phenotype, then applied Algorithm 1 on this synthetic data set to tag the SNPs. Here we recognized as a tag SNP the top-ranked SNP of the attractor which has the largest 3rd weight among the attractors found using seeds in the genomic region. The association between a SNP and the phenotype is analyzed using the *χ*^2^ test. The analysis indicates that the meta SNP method identified one of the designated SNP as the tag SNP and assigned the 2nd and 3rd highest weights to the other two designated SNPs. The association of these three SNPs and the phenotype are significant (S3 Table), which suggests that the metaSNP method correctly tagged the designated SNPs.

### Complexity

**Fig 9:**
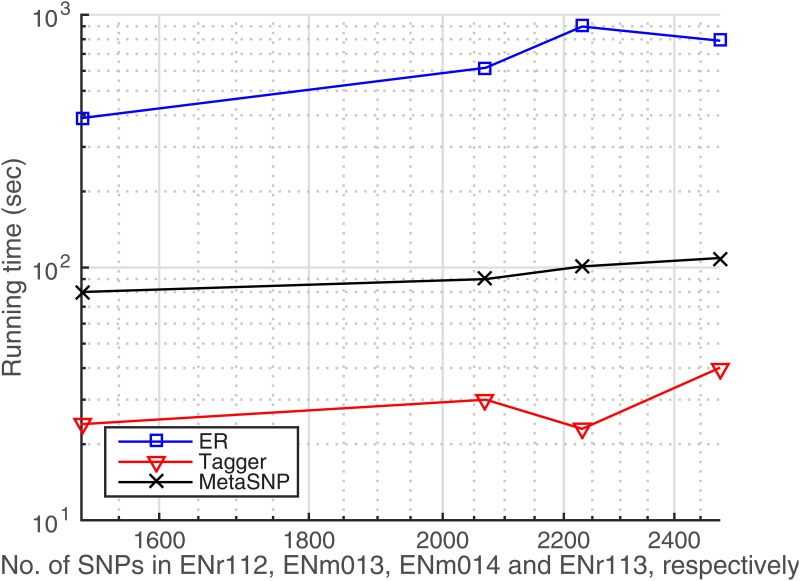
Running times in imputed genotypes data tested in ENCODE regions, HapMap.

**Fig 10:**
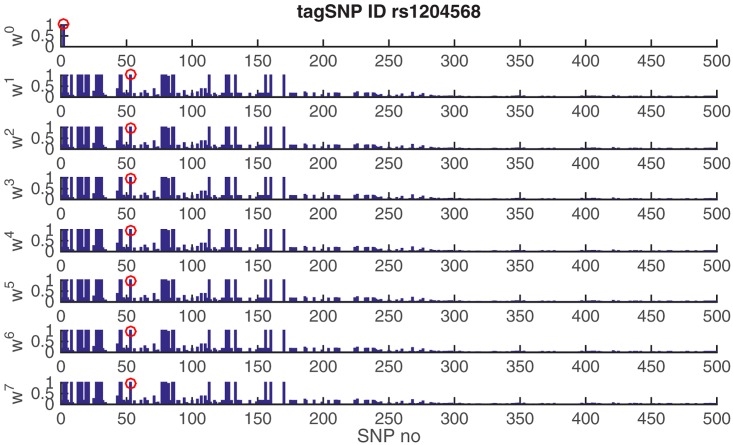
Convergence of the algorithm for an estimated attractor. Each panel displays the weight vector ***w*** calculated at an iteration as defined after the eq (1). The vertical axis displays the weights and the horizontal axis the SNPs in the order of genomic locations. The tag SNP is marked by the red circle through the iterations ***w***^0^, …, ***w***^7^, which is selected according to its proximity to the genomic median of the co-top-ranking SNPs. Only the first-500 SNPs in the ENCODE’s ENr113 region are displayed.

The computational complexity of algorithms is evaluated in terms of running times in the benchmark HapMap data. We carried out all experiments on a hardware with Core(TM) i7 CPU @2.6GHz, 16GB memory, and Mac OSX 10.10.5. The pairwise LD-based Tagger is the fastest approach in this study as it can process each ENCODE data set in a minute (Fig 9). On the other hand, the multi-locus LD calculation substantially slows down the ER algorithm, although it results in a constant growth of complexity increasing with the data size. MetaSNP’s performance is comparable to Tagger and seems to be unaffected from the data size as well, displaying a log-linear behavior which is desirable for a block-free approach based on the multi-locus analyses of SNPs. On the other hand, for most attractors the metaSNP algorithm converges in less than 20 iterations. Fig 10 displays the convergence of the algorithm in 7 iterations when it estimates the attractor from the second seed and its tag SNP (*rs1204568*) in the ENm014 genotypes data. In this figure, we see that the tag SNP was accurately predicted in the first iteration (*w*^1^) and lasted to the last iteration (*w*^7^) given the threshold *ϵ* = 1*E* − 7, which presents that the algorithm can efficiently converge within the few iterations.

#### Complexity in tagging the 1000 Genomes genotypes

The genome-wide application of the metaSNP algorithm (*step* 1 and *step* 2 in Algorithm 2) is run with the cluster support:

In *step* 1, the scanning for the “short-attractors” were run on 100 m1.medium instances on Amazon Web Services using StarCluster [47] and R. [48]. Each instance is equipped with a vCPU (Intel Xeon Family) and 3.75 GB RAM. In *step* 2, the refinement of the “short-attractors” were run on a workstation with Dual Intel Xeon E5-2637 (16 cores) and 256GB RAM using R.

For the whole genome, the total running times for *step* 1 and *step* 2 are 134.37 hours and 153.3 hours, respectively.

## Discussion

This study addresses the problem of efficient SNP tagging in very large genomic distances by exploiting the underlying multi-locus LD patterns. We emphasize that our SNP tagging approach is block-free and undemanding in the sense that it is not based on any prior information derived from the empirical data such as haplotype block structures, recombination hotspots, or LD maps. In contrast, although the other methods that used the distance measures that are closely related to the mutual information [16, 17], they require the prior assumptions on the blocks and optimization parameters to select the tag SNPs in a two-phased framework. The proposed method requires only the genotype (or haplotype) sequences (i.e., trilevel or twolevel information of allelic variation) to perform SNP tagging, and offers a number of parameters to provide flexibility for different genotyping needs. For example, the different values of *α* could be chosen for optimizing different objectives of attractor searching: in Algorithm 1 we used *α* = 2 in *step* 1 for obtaining all valuable short-attractors even with low degrees of LD at the expense of getting highly-correlated (redundant, overlapping) ones; then in *step* 2, as opposed to that, we used *α* = 5 to focus on only the sharpest (distinctive) attractors. Further, the algorithm can be started with a user-defined SNP set as the “primary seeds” to be force-included in the final results. In this case, the *α* parameter can be accordingly adjusted (i.e., increased) for those seed SNPs to ensure the discovery of “sharp” attractors that mutually exclude each other’s seed SNP.

Since the objective is based on a general measure of correlation between the variables (i.e., SNP loci) the employed algorithm can handle phased haplotype sequences as well as the genotype data. This is a great advantage over existing algorithms since the procedures that apply statistical inference on data management (e.g., haplotype phasing, genotype imputation etc.) is prone to error and often introduce dependencies and arbitrary patterns to the end results, whereby algorithms capable of handling different data types can avoid such limitations. This allows the flexibility for analyzing multiple data sets in a study, which re-phasing all the data sets using the same method may be infeasible as well as scrutinizing the pipeline of analysis.

As a measure of multi-locus correlation we used the mutual information that can be efficiently estimated between a continuous valued metaSNP variable and an individual SNP variant. For this, we employed the numerical method in [49] which is based on partitioning the continues data into discrete intervals (bins) and assigning each data into several bins simultaneously by the use of B-spline functions. For computational efficiently, we used 4 bins which effectively capture the variation in trilevel data, and the spline order of 2 to allow the continues data to be represented by two bins at most. The details of the mutual information estimation is given in the S1 File.

In addition to the ranked tag SNPs, the proposed method reports their metaSNPs and the attractors representing the association landscapes, providing an information-rich output for subsequent analyses. An example output corresponding to ENr113 genotypes results is given in S2–S4 Figs and in the Supplemental Data (S4 Table) as well. The metaSNP and the converged attractor captures the underlying haplotype structure in several layers, where a number of top-ranking SNPs selected by a specific weight threshold can represent a haplotype block of particular degree of LD.

The main contributions of the proposed algorithm are the definitions of “attractor” and “metaSNP” for the simplified representations of “multi-locus LD” and “variation” in the haplotype blocks, respectively, which may provide valuable information in both dimensions of the SNP data. Furthermore, these quantities can be iteratively estimated up to a certain precision, allowing efficient heuristic designs which can be applied to very large data set. In particular, our sliding-window heuristic can employ the vast amount of information in several layers, i.e., using the first layer of data (sliding a 101-SNPs window) we find all potential attractee seeds that may form a typical attractor, then using the second layer of data (the window of 10,001-SNPs local to a given seed) we find the desired long attractors to capture any long-range multi-locus LD pattern. Since the SNP variability is not uniform across a chromosome [50], the distribution of the numbers of tag SNPs that are required for a high coverage rates of each of the chromosomes may be different from the numbers for short regions with different SNP densities.

In this study, we defined the genomic neighborhood of a tag SNP as the set of X = 10,001 nearby variant that will exhibit a substantial LD decay in the corresponding genomic distance (i.e., 500kb region). Although in 1KGP data sets a large drop in pair-wise LD (*r*^2^ < 0.05) is observed within the 100 kb distance in all populations [42], we extend our LD decay assumption to ∼500 kb (i.e., 10,001 SNPs) since we use a different LD score (the similarity metric *J*) to be able to capture the multi-locus associations that might be (jointly) present in larger distances. Because the density of SNP varies across the genome and metaSNP algorithm automatically determines the size of a haplotype, we used a sliding window of 10000 SNPs to recruit a consistently large number of SNPs within a genomic region for the algorithm to identify the haplotype within a reasonable time. This also provides an opportunity for analyzing the structure of the associations of variants and reveal the properties of the “long-range” correlations of variants as the correlation of DNA sequences, which will be an interesting topic in the future study [51].

## Conclusion

In summary, we have presented a new block-free approach for solving the genome-wide SNP tagging problem which only requires the genomic sequence data. The employed algorithm is by nature block-free and discovers the joint associations between the variants based on a measure of multi-locus mutual information. Experimental results indicate that the metaSNP approach can efficiently find tag SNPs covering a greater majority of genomic diversity in comparison to existing algorithms. It outperforms the relatively faster pairwise-LD based Tagger in all data sets from the HapMap and the 1000 Genomes Project, achieving a better balance between coverage and genotyping cost, and efficiently scales up to genome-wide analyses. In the relevant haplotype experiments, metaSNP significantly outperforms the multi-locus LD based ER algorithm in terms of both coverage-cost balance and computational complexity. Extensions to missing data tests are also carried out and the algorithm is shown to be robust in such conditions. In particular, we performed a novel application to 1000 Genomes Project data and produced a reference resource of tagging variants with detailed descriptions of associations to the SNPs they tag. Through rigorous tests, we observed that a small set of ∼15-20 tag SNPs per chromosome can represent the genetic diversity of thousands of (multi-population) samples, which is a more cost-effective solution compared to Tagger’s estimates on the same data sets. In default settings, the metaSNP approach yields a proper tradeoff between the amount of required genotyping and coverage rate, offers useful parameters to fulfill requirements in different applications, is versatile to perform on different data types, and produces rich output for subsequent association studies.

## Supporting Information

S1 Fig Locations of the tag SNPs in the Chromosomes 21 and 22 in the 1000 Genomes Project database. Details are given in S5 Table.
(PDF)

S1 Table Statistical tests on HapMap results. The performances of the two tagging methods in Tables 2 and 4 is tested by using the z-test as well as McNemar’s test. For Table 3, the difference of the tagging methods is tested by t-test. The Kolmogorov-Smirnov tests for the Figs 2–5 are also included.
(XLS)

S2 Table Comparison of the tag SNP sets identified in HapMap ENCODE regions (Fig 2). The exclusive and intersecting sets of tag SNPs that are found by the two algorithms, metaSNP and Tagger.
(XLS)

S2 Fig Attractor metaSNP results obtained from the ENr113 genotypes in HapMap database. Multi-locus mutual similarity (LD) estimates are displayed in the estimated attractors that correspond to the discovered tag SNPs, top-10.
(PDF)

S3 Fig Attractor metaSNP results obtained from the ENr113 genotypes in HapMap database. Multi-locus mutual similarity (LD) estimates are displayed in the estimated attractors that correspond to the discovered tag SNPs, top 11-20.
(PDF)

S4 Fig Attractor metaSNP results obtained from the ENr113 genotypes in HapMap database. Multi-locus mutual similarity (LD) estimates are displayed in the estimated attractors that correspond to the discovered tag SNPs, top 20-27.
(PDF)

S3 Table Phenotype association tests. Tagging performance of the proposed method through the comparison of genotype-phenotype association of the tagged SNP and the SNPs that were correlated with the tagged SNP.
(XLS)

S4 Table Data corresponding to S2–S4 Figs. Each of the 27 sheets correspond to an estimated attractor, given in the same order as supplementary figures. For convenience, only the data for the 100 top-ranking SNPs are displayed, corresponding to the SNP information, the estimated mutual information weights, and the trilevel genotype values.
(XLS)

S5 Table Data corresponding to tag SNPs selected in each analysis. Detailed information of the tag SNPs selected by each algorithm from the analyzed data bases, corresponding to Figs 2–7.
(XLS)

S1 File Supplementary Material. Detailed description of the mutual information model used for the attractor metaSNP algorithm.
(PDF)
