# Supplementary figures and images for: Discovering Genome-Wide Tag SNPs Based on the Mutual Information of the Variants
Source: PLoS One. 2016 Dec 16;11(12):e0167994. doi: 10.1371/journal.pone.0167994 (PMC5161470; doi:10.1371/journal.pone.0167994)

21

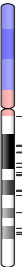

■ chr21\_tagSNPs.bed

22

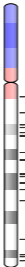

■ chr22\_tagSNPs.bed

Supplement: S1 Fig — Details are given in S5 Table. (PDF) [file pone.0167994.s001.pdf]

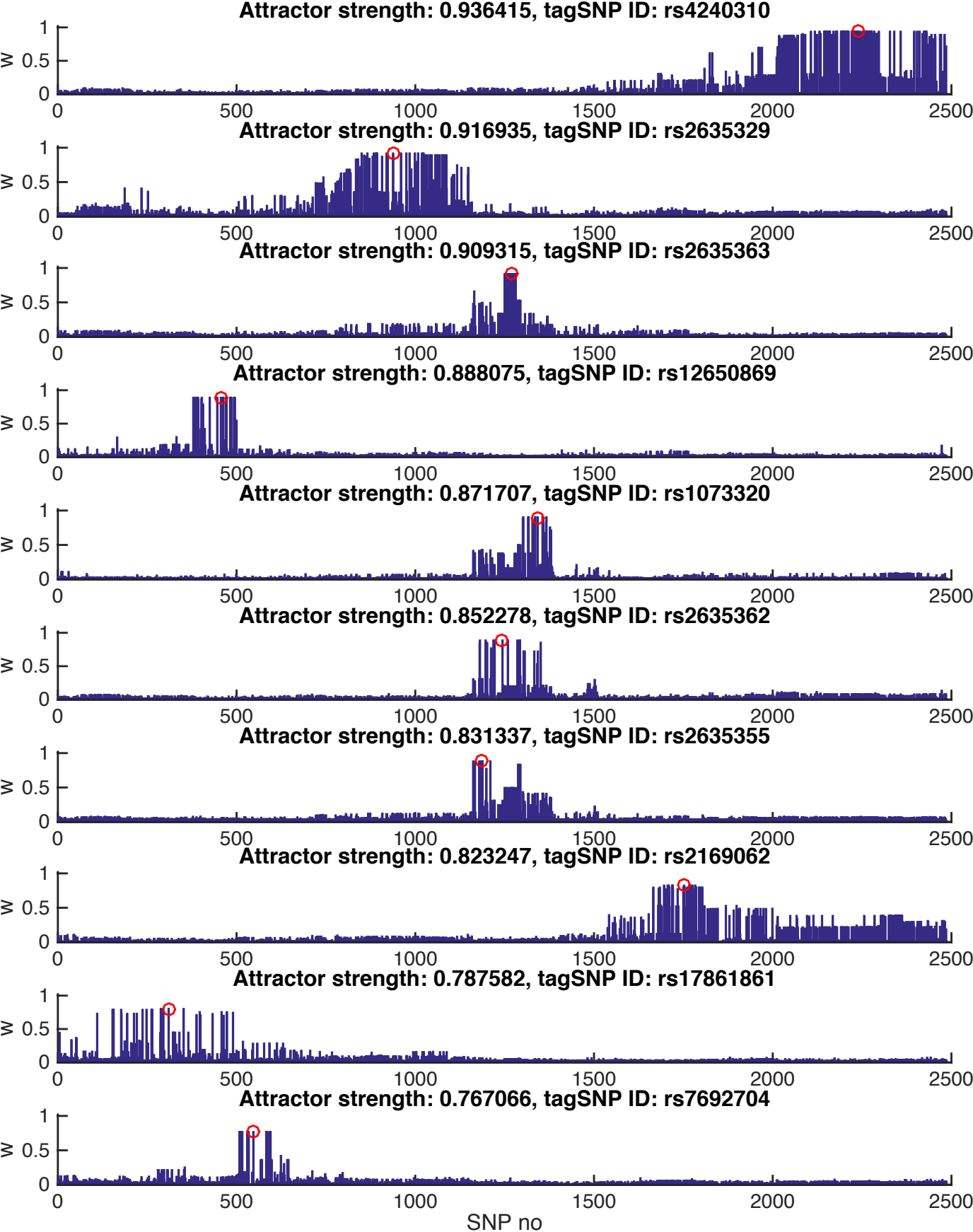

Supplement: S2 Fig — Multi-locus mutual similarity (LD) estimates are displayed in the estimated attractors that correspond to the discovered tag SNPs, top-10. (PDF) [file pone.0167994.s004.pdf]

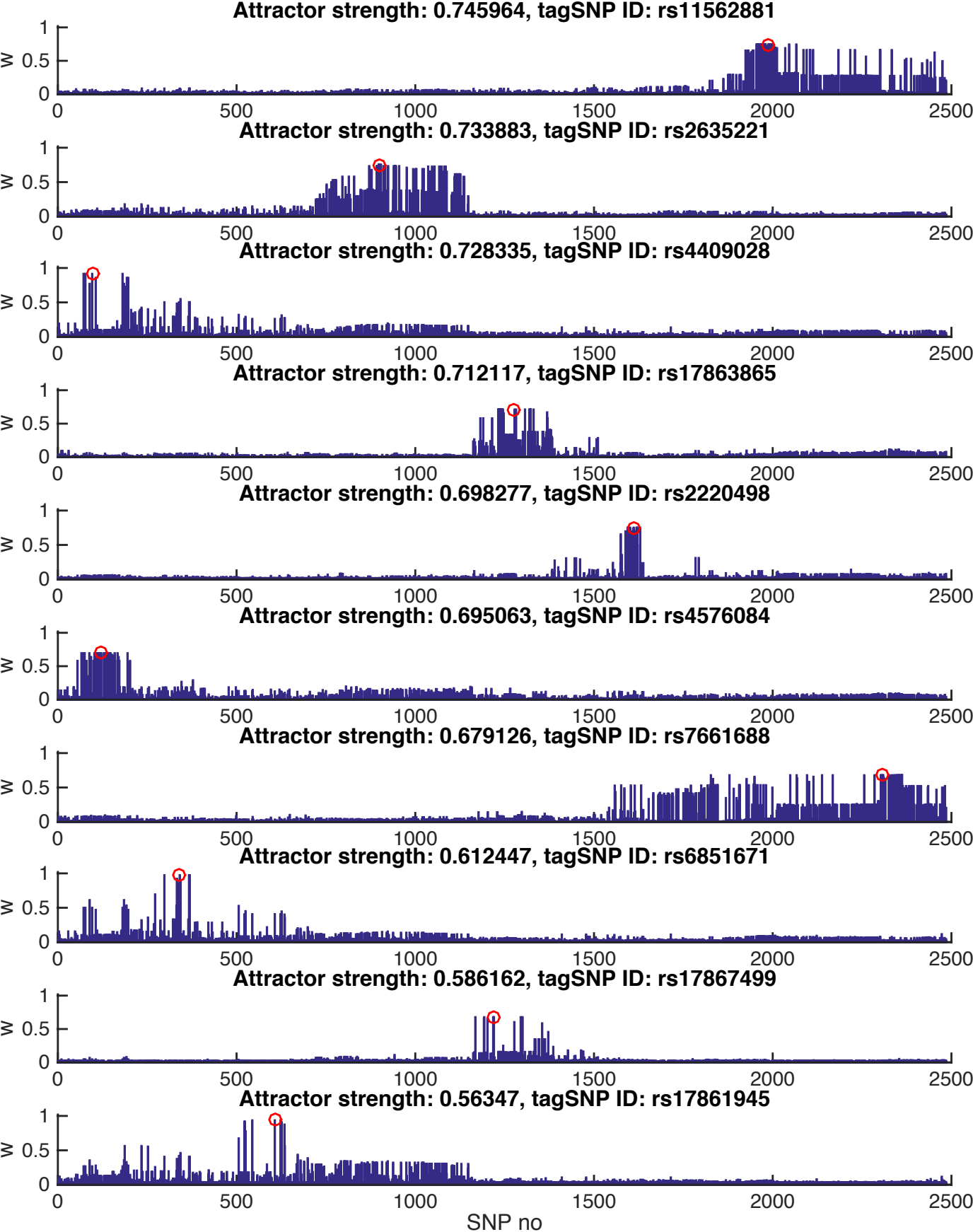

Supplement: S3 Fig — Multi-locus mutual similarity (LD) estimates are displayed in the estimated attractors that correspond to the discovered tag SNPs, top 11-20. (PDF) [file pone.0167994.s005.pdf]

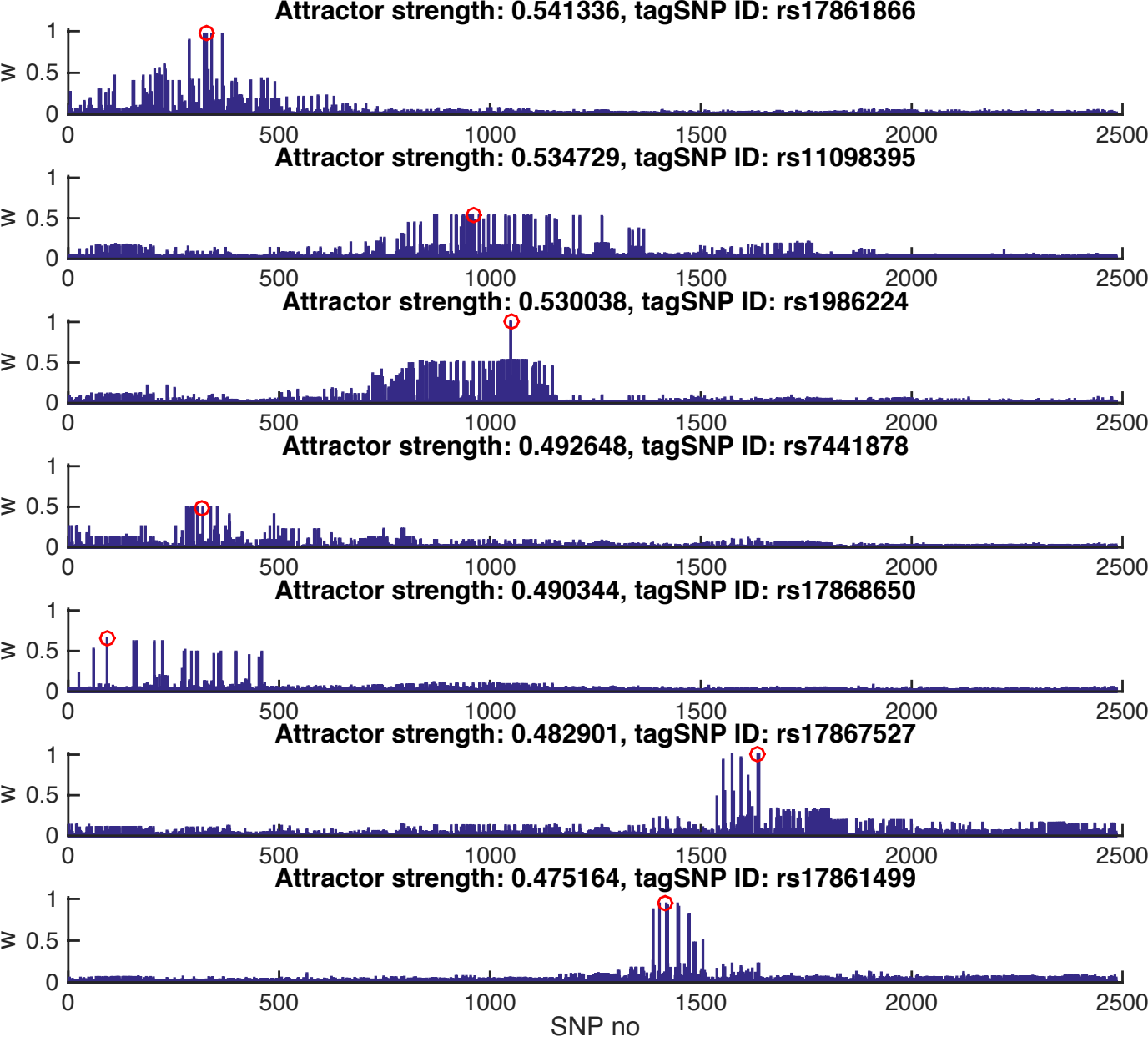

Supplement: S4 Fig — Multi-locus mutual similarity (LD) estimates are displayed in the estimated attractors that correspond to the discovered tag SNPs, top 20-27. (PDF) [file pone.0167994.s006.pdf]
