# Supplementary material for: Discovering Genome-Wide Tag SNPs Based on the Mutual Information of the Variants
Source: PLoS One. 2016 Dec 16;11(12):e0167994. doi: 10.1371/journal.pone.0167994 (PMC5161470; doi:10.1371/journal.pone.0167994)
Supplement: S1 File — Detailed description of the mutual information model used for the attractor metaSNP algorithm. (PDF) [file pone.0167994.s010.pdf]

DISCOVERING GENOME-WIDE TAG SNPS BASED ON THE MUTUAL INFORMATION OF THE VARIANTS

SUPPLEMENTARY MATERIAL

Mutual Information Estimation

Mutual information is a measure of statistical dependence between two variables X, Y. Initially developed for categorical variables, it quantifies the information about one random variable through the other random variable, i.e.,

$$I(X,Y) = \sum_{x \in X} \sum_{y \in Y} p(x,y)\ log \frac{p(x,y)}{p(x)p(y)} \tag{1}$$

where $p(x)$ is the probability (frequency) of observing the state (category) x in the variable X, and similarly $p(x,y)$ is the joint probability of observing the state (x,y) together in the variables X and Y, respectively.

To calculate mutual information for two continuous variables, the continues values should be quantized into discrete intervals (making them categorical variables), thereby the probabilities (frequencies) of each discrete state (interval) can be measured. For example, given N measurements of the variable X, i.e., $x_n \in \mathcal{R}, n = 1, \ldots, N$, an indicator function Θ_i counts the number of data points falling into each interval (bin) $b_i, i = 1, \ldots, M$, then the probabilities are estimated as the relative frequency of occurrences of the bins

$$\hat{p}_X(b_i) = \frac{1}{N} \sum_{n=1}^{N} \Theta_i(x_n)$$

where $\Theta_i(x_n) = 1$ if $x_n \in b_i$, and $\Theta_i(x_n) = 0$ otherwise. The joint probabilities $\hat{p}_{X,Y}(b_{i,j})$ are calculated analogously from the multivariate extension, then using the equation (1) the mutual information is calculated given those probability estimates.

However, assigning each data point to only one bin may have limitations, as some data points appearing near the border of two bins may be shifted towards the neighboring bin due to noise in the data, and this can significantly effect the mutual information estimate. To deal with this, the same data point may be assigned to multiple bins simultaneously. A proper assignment function Θ_i may be a set of polynomial *B-spline* functions [1], where

the spline order (k) defines the number of bins each data point will be assigned to. The k-th order *B-spline* functions $B_{i,k},\ i = 1, \dots, M$ can be recursively defined as [2]

$$B_{i,1}(z) = \begin{cases} 1 & \text{if} \quad t_i \leq z < t_{i+1} \\ 0 & \text{otherwise,} \end{cases}$$

$$B_{i,k}(z) = B_{i,k-1}(z)\frac{z - t_i}{t_{i+k-1} - t_i} + B_{i+1,k-1}(z)\frac{t_{i+k} - z}{t_{i+k} - t_{i+1}},$$

where $z \in [0, M - k + 1]$ represents the interval, and t_i is the knot vector defined for a given number of bins $i = 1, \dots, M$ and a given spline order k

$$t_i = \begin{cases} 0 & \text{if} \quad i < k \\ i - k + 1 & \text{if} \quad k \leq i \leq M - 1 \\ M - k + 1 & \text{if} \quad i > M - 1. \end{cases}$$

In our study, we estimate the mutual information by using the aforementioned *B-spline* based method which was introduced in [2]. We distribute the continuous values appearing in a *metaSNP* into $M = 4$ bins, and use the spline order $k = 2$ whereby each value is assigned to at most 2 bins. We experimentally observed that the choice of $M = 4$ bins is sufficient to represent the trilevel genotype information, and do not compromise performance compared to that of using a larger number of bins ($M = 6$). For elaborate presentation of the *B-spline* algorithm we refer readers to [2]. For further detail on the *B-spline* functions we refer to [3, 4].

We aim to find a proper mutual similarity metric between a SNP s and the metaSNP $\mathcal{M}$. Once we estimate their mutual information $I(s, \mathcal{M})$ by using the above method, we normalize this non-negative measure by dividing the maximum of the individual measures $I(s, s)$, and $I(\mathcal{M}, \mathcal{M})$ to bound the maximum possible value by 1. Then we impose a power exponent to shape this normalized measure in a nonlinear manner pushing smaller values closer to zero, i.e.,

$$J(s_i, \mathcal{M}) = I^{\alpha}(s_i, \mathcal{M}),$$

where α is a non-negative constant. A larger α forces the algorithm to find a "sharper" (more distinctive) attractor where the majority of the mutual similarity is concentrated to the fewer top-SNPs. We experimentally set $\alpha = 5$ as it yielded good performance across various data sets.

References

[1] Stoyan G. de Boor, C., A Practical Guide to Splines. Applied Mathematical Sciences 27. Berlin-Heidelberg-New York, Springer-Verlag 1978. XXIV, 392 S. ZAMM, Journal of Applied Mathematics and Mechanics, Zeitschrift fur Angewandte Mathematik und Mechanik. 1980;60(1):62–62. doi:10.1002/zamm.19800600129.

[2] Daub CO, Steuer R, Selbig J, Kloska S. Estimating mutual information using B-spline functions –an improved similarity measure for analysing gene expression data. BMC Bioinformatics. 2004;5:118–118. doi:10.1186/1471-2105-5-118.

[3] Unser M, Aldroubi A, Eden M. B-spline signal processing. I. Theory. IEEE Transactions on Signal Processing. 1993;41(2):821–833. doi:10.1109/78.193220.

[4] Unser M, Aldroubi A, Eden M. B-spline signal processing. II. Efficiency design and applications. IEEE Transactions on Signal Processing. 1993;41(2):834–848. doi:10.1109/78.193221.
